# Microstructural and Cerebral Blood Flow Abnormalities in Subjective Cognitive Decline Plus: Diffusional Kurtosis Imaging and Three-Dimensional Arterial Spin Labeling Study

**DOI:** 10.3389/fnagi.2021.625843

**Published:** 2021-02-01

**Authors:** Zhongxian Yang, Yu Rong, Zhen Cao, Yi Wu, Xinzhu Zhao, Qiuxia Xie, Min Luo, Yubao Liu

**Affiliations:** ^1^Medical Imaging Center, Shenzhen Hospital, Southern Medical University, Shenzhen, China; ^2^Medical Imaging Center, The Second Affiliated Hospital, Medical College of Shantou University, Shantou, China; ^3^Department of Neurology, The People's Hospital of Gaozhou City, Maoming, China; ^4^Department of Neurology, Shantou Central Hospital and Affiliated Shantou Hospital of Sun Yat-sen University, Shantou, China

**Keywords:** diffusional kurtosis imaging, arterial spin labeling, subjective cognitive decline plus, mild cognitive impairment, Alzheimer's disease

## Abstract

**Objective:** To explore microstructural and cerebral blood flow (CBF) abnormalities in individuals with subjective cognitive decline plus (SCD plus) using diffusional kurtosis imaging (DKI) and three-dimensional (3D) arterial spin labeling (ASL).

**Methods:** Twenty-seven patients with SCD plus, 31 patients with amnestic mild cognitive impairment (aMCI), and 33 elderly controls (ECs) were recruited and underwent DKI and 3D ASL using a GE 3.0-T MRI. Mean kurtosis (MK), fractional anisotropy (FA), mean diffusivity (MD), and CBF values were acquired from 24 regions of interest (ROIs) in the brain, including the bilateral hippocampal (Hip) subregions (head, body, and tail), posterior cingulate cortex (PCC), precuneus, dorsal thalamus subregions (anterior nucleus, ventrolateral nucleus, and medial nucleus), lenticular nucleus, caput nuclei caudati, white matter (WM) of the frontal lobe, and WM of the occipital lobe. Pearson's correlation analysis was performed to assess the relationships among the DKI-derived parameters, CBF values, and key neuropsychological tests for SCD plus.

**Results:** Compared with ECs, participants with SCD plus showed a significant decline in MK and CBF values, mainly in the Hip head and PCC, and participants with aMCI exhibited more significant abnormalities in the MK and CBF values than individuals with ECs and SCD plus in multiple regions. Combined MK values showed better discrimination between patients with SCD plus and ECs than that obtained using CBF levels, with areas under the receiver operating characteristic (ROC) curve (AUC) of 0.874 and 0.837, respectively. Similarly, the AUC in discriminating SCD plus from aMCI patients obtained using combined MK values was 0.823, which was also higher than the combined AUC of 0.779 obtained using CBF values. Moreover, MK levels in the left Hip (h) and left PCC positively correlated with the auditory verbal learning test-delayed recall (AVLT-DR) score in participants with SCD plus. By contrast, only the CBF value in the left Hip head positively correlated with the AVLT-DR score.

**Conclusions:** Our results provide new evidence of microstructural and CBF changes in patients with SCD plus. MK may be used as an early potential neuroimaging biomarker and may be a more sensitive DKI parameter than CBF at the very early stage of Alzheimer's disease (AD).

## Introduction

Subjective cognitive decline (SCD), the first clinical manifestation in the Alzheimer's disease (AD) continuum, refers to a self-experienced cognitive capacity decline and has been shown to be associated with a high risk of conversion to AD (Tandetnik et al., 2015). As the cognitive function in patients with SCD is within normal limits, most neuropsychological evaluations find it difficult to capture the subtle cognitive decline, especially memory loss. Patients with SCD are twice as likely to develop mild cognitive impairment (MCI) than those without SCD (Mitchell et al., 2014). Previous studies have also shown that SCD patients present with atrophy in the hippocampus (Hip), the paraHip, the medial temporal, and the frontoparietal gray matter (GM) (van der Flier et al., 2004; Saykin et al., 2006; Wen et al., 2019); alterations in the white matter (WM) (Song et al., 2016); decline of brain metabolism (Jeong et al., 2017); accumulation of high β-amyloid (Aβ) (Snitz et al., 2013); and disruption of functional activity (Sun et al., 2016). These results showed that SCD, MCI, and AD are regarded as a spectrum of clinical disorders (Kiuchi et al., 2014; Yan et al., 2018; Reisberg et al., 2020). However, SCD may also be caused by many other factors in addition to the pathophysiology underlying AD, such as psychological factors, drug use, and other medical or neurological conditions (Perrotin et al., 2017). SCD plus, which leads to a higher risk of AD than SCD, may be a very early stage of AD that precedes amnestic MCI (aMCI). The features of SCD plus include (Jessen et al., 2014) subjective decline limited only to memory rather than other cognitive domains, complaints of SCD within the last 5 years, an age at onset of SCD ≥ 60 years, concerns (memory loss) associated with SCD, complaints of feeling worse than others in the same age group, confirmation of cognitive decline by an informant, and the presence of the apolipoprotein E-ε4 (APOE-ε4) genotype and other biomarker evidence for AD. Thus, identifying neuroimaging biomarkers for SCD plus is essential for early detection, early intervention, and reduction of the burden of dementia in the population.

Diffusion kurtosis imaging (DKI), developed based on diffusion tensor imaging (DTI), can provide additional metrics related to the non-Gaussianity of water diffusion, such as mean kurtosis (MK), axial kurtosis, and radial kurtosis (Jensen et al., 2005). Diffusion parameters, such as fractional anisotropy (FA) and mean diffusivity (MD), can also be obtained from DKI. As one of the main parameters of DKI, MK has been used to describe the microstructural complexity or heterogeneity of the tissue. It can assess the microstructure of both WM and GM, particularly GM (Jensen et al., 2005). FA is mostly used for assessing WM fiber tract integrity, and MD can assess microstructural alterations of GM and WM. DKI has shown great potential in diagnosing several disorders of the nervous system, such as neurodegenerative disease, brain infarction, gliomas, and mental illness (Guo et al., 2016; Zhao et al., 2016; Guan et al., 2019; Haopeng et al., 2020; Huang et al., 2020; McKenna et al., 2020). Previous studies have also reported that, in patients with MCI and AD, DKI can detect microstructural alterations in GM (Falangola et al., 2013; Gong et al., 2013; Struyfs et al., 2015; Chen et al., 2017), as well as in WM (Yuan et al., 2016; Gong et al., 2017; Song et al., 2019). Regarding GM and the subcortical nucleus, one study found that subjects with aMCI showed significant MK abnormalities in many regions, including the bilateral Hip, the thalamus, the putamen, and the globus pallidus. MD revealed the second most significant changes of the DKI parameters (Gong et al., 2017). Another study showed that MK and FA could detect microstructural complexities, such as the globus pallidus, the substantia nigra, and the red nucleus, in healthy participants (Gong et al., 2014). Thus, estimating microstructural changes related to GM and the subcortical nucleus is very important to improve the early diagnosis of AD. However, no report has investigated SCD plus-related DKI regarding microstructural alterations. Arterial spin labeling (ASL) is a valuable non-invasive imaging tool that is used to magnetically label arterial blood water as an endogenous contrast medium tracer to quantify cerebral blood flow (CBF; Alsop et al., 2000). Compared with PET imaging, the major advantages of ASL are its lower costs, its non-invasiveness, and its reduced scan time (Henriksen et al., 2012), particularly at 3.0 Tesla (T) (Yoshiura et al., 2009). As the technology underlying MRI hardware and software continues to improve, ASL-measured CBF is becoming an increasingly widely available method for distinguishing MCI/AD from elderly controls (ECs) with comparable accuracy to PET (Haller et al., 2016; Fällmar et al., 2017; Riederer et al., 2018). The most consistent findings in ASL studies from patients with MCI and AD have revealed brain hypoperfusion mainly in the posterior cingulate cortex (PCC) and the precuneus (Pr), as well as in the bilateral parietal and temporal areas. Many previous studies have described the association of changes and vulnerable regions with MCI/AD [including Hip, PCC, Pr, dorsal thalamus (DT), nuclei basales, WM of the frontal lobe (FLWM) and WM of the occipital lobe (OLWM), particularly Hip, PCC, and Pr] by structural MRI, DTI, magnetic resonance spectroscopy, and functional MRI (Pennanen et al., 2004; De Jong et al., 2008; Van Straaten et al., 2008; Jahng et al., 2011; Yang Z. X. et al., 2012). These findings suggest that MCI and/or AD are caused by damage not to a single brain area but to multiple areas of the brain. With advanced MRI techniques, DKI and ASL may supply microstructural and hemodynamic information at the early stage of AD. Additionally, the underlying mechanism regarding which imaging metrics (MK, FA, MD, and CBF) changed first in subjects with SCD plus must be precisely elaborated. Furthermore, changes in CBF in subjects with SCD plus are rarely reported, particularly when combined with substructural measures. To our best knowledge, the diagnostic performance of DKI and ASL in assessing brain microstructural and CBF alterations in subjects with SCD plus has not been investigated.

To enhance our knowledge of the underlying dementia processes, facilitate an early diagnosis, guide clinical trials, and understand the characteristics of the pathophysiological basis of the SCD plus stage, in the current study, we aimed to evaluate brain microstructural and CBF changes in patients with SCD plus using DKI and three-dimensional (3D) ASL imaging measured in multiple brain regions to determine how these regions are altered in this stage. Additionally, we also assessed the relationships between DKI-derived parameters, CBF values, and key neuropsychological test scores among patients with SCD plus.

## Materials and Methods

### Human Subjects and Neuropsychological Testing

Ninety-one participants were recruited for this study from September 2017 to July 2019, including 27 patients with SCD plus, 31 patients with aMCI, and 33 age- and sex-matched ECs. The SCD plus and aMCI groups were recruited from the outpatient clinic, and ECs were recruited through a medical examination center. Before enrollment, written informed consent was obtained from all participants or their legal guardians. The study was approved by the Ethical Committee of Second Affiliated Hospital of Shantou University Medical College (Registration No. 2017-10), and all procedures were performed in accordance with the Declaration of Helsinki. All participants provided their demographic and clinical data (including age, sex, education, and living conditions) and had undergone cognitive evaluations by two expert neurologists. All participants had also undergone a series of standardized neuropsychological evaluations, including tests that measured cognitive functioning in the domains of memory, executive functioning, attention, and language. The basic set of psychological tests included the Mini-Mental State Examination (MMSE; Folstein et al., 1975), the Montreal Cognitive Assessment (MoCA, Beijing version; Lu et al., 2011), the Clinical Dementia Rating (CDR) Scale (Morris, 1993), the Global Deterioration Scale (GDS; Reisberg et al., 1982), Chinese Huashan version of auditory verbal learning test [AVLT, including AVLT-immediate recall, AVLT-delayed recall (AVLT-DR), and AVLT-recognition; Xu et al., 2020], activities of daily living assessment (Barberger-Gateau et al., 1992), the Hachinski Ischemic Scale (HIS; Larson et al., 1989), and the Hamilton Depression Rating Scale (HAM-D; Worboys, 2013).

Patients with SCD plus met the following criteria proposed by the SCD Initiative (SCD-I; Jessen et al., 2014): (a) self-reported persistent cognitive complaints of memory decline ≤ 5 years; (b) confirmation of cognitive decline by an informant; (c) onset age ≥ 60 years; (d) feeling worse than peers of the same age and concerns associated with SCD; (e) performance within normal limits for age and educational attainment on the MMSE, MoCA, and ADL after sex, age, and education adjustment; (f) a GDS score of 2; (g) a CDR score of 0; and (h) a HIS score <4. Patients with aMCI met the criteria defined by Petersen et al. (2001) as follows: (a) memory decline according to clinical judgment or confirmed by an informant; (b) objective decline of episodic memory, determined by the neurologists' judgment based on a neuropsychological evaluation; (c) normal general cognitive function determined by a CDR score of 0.5, a GDS score of 3, a HIS score <4, and an MMSE ≥ 24; and (d) ineligibility for AD according to the criteria of the National Institute of Neurological and Communicative Diseases and Stroke-Alzheimer's Disease and Related Disorders Association. ECs were defined as those without subjective cognitive complaints, without physical, psychiatric, or neurological disorders, without abnormal findings on conventional brain MRI, and with normal performance on neuropsychological tests. The exclusion criteria for all participants included the following: (a) a HIS score > 4; (b) a HAM-D score > 24; (c) specific causes of WM lesions, such as multiple sclerosis, epilepsy, encephalitis, tumors, cranial arteritis, and trauma; (d) AD, Lewy body dementia, frontotemporal dementia, or Parkinson's disease; (e) intracranial hemorrhage, Moyamoya disease, aphasia, hepatic encephalopathy, systemic diseases, and signs of normal pressure hydrocephalus or carbon monoxide poisoning; and (f) alcohol dependence and other psychoactive substance abuse history, serious medical disease, or mental illness.

### Conventional MRI, DKI, and ASL Data Acquisition

Conventional MRI scans were acquired using a standard quadrature eight-channel head coil with a 3.0T MRI scanner (Signa HDx Twin speed; GE Medical Systems, Milwaukee, Wisconsin, USA). Next, we changed the host scanning method from the clinical mode to the research mode. Using the research mode, DKI scanning parameters were obtained using an echo-planar imaging (EPI) sequence as follows: repetition time (TR)/echo time (TE) = 6,000 ms/109 ms; diffusion gradient pulse duration = 32.2 ms; diffusion gradient separation = 38.8 ms; slice thickness = 4 mm; slice gap = 0 mm; field of view (FOV) = 240 × 240 mm; matrix = 256 × 256; number of excitations (NEX) = 1; *b*-values = 0, 1,000, 2,000 s/mm^2^; diffusion-encoding directions = 15; number of slices = 24; and scanning time = 250 s. 3D pseudocontinuous ASL images were acquired using the following scanning parameters: TR/TE = 4,580/9.8 ms; FOV = 240 × 240 mm; slice thickness = 4 mm; slice gap = 0 mm; postlabeling delay time = 1,500 ms; and scanning time = 266 s. We used the oblique sagittal position as the scanning standard. The bottom of the slab was positioned at the bottom of the cerebellum, and the top of the slab was positioned at the top of the semioval center, with coverage of mostly the cerebrum. Cushions and cotton balls were used to reduce subject movement and scanner noise.

### Image Post-processing and Data Analysis

All raw DKI images were transferred to a GE Advantage Workstation version 4.6 (GE Medical System, Rue de la Minière, France) and post-processed in the FuncTool software package 9.0 environment. Before DKI parametric maps were generated, all raw data were automatically performed with the affine and rigid body registrations to the B0 image for reducing EPI distortion, eddy-current distortion, and head motion. Next, the DKI parameters, including MK, FA, and MD, were automatically generated by the DKI software, which was developed using the Applied Science Laboratory of GE (http://www.nitrc.org/projects/dke/). Quantitative MK, FA, and MD maps were generated from the DKI model with *b*-values = 0, 1,000, and 2,000 s/mm^2^ by the following equation:

$$\begin{array}{l} \text{In}\left[S_{\left(n,b\right)}/S_{0}\right]=-b\overset{3}{\underset{i=1}{\sum }}\overset{3}{\underset{j=1}{\sum }}n_{i}n_{j}D_{ij}\\ \;+\frac{1}{6}b^{2}D^{-2}\overset{3}{\underset{i=1}{\sum }}\overset{3}{\underset{j=1}{\sum }}\overset{3}{\underset{k=1}{\sum }}\overset{3}{\underset{l=1}{\sum }}n_{i}n_{j}n_{k}n_{l}W_{ijkl} \end{array}$$

*S*_(*n*__, *b*)_ represents the diffusion encoding direction *n* and diffusion signal intensity for diffusion weighting *b*, and *S*_0_ represents the signal intensity for minimally diffusion-weighted imaging (*b*_0_)_._ *D*_*ij*_ and *W*_*ijkl*_ represent the components of the diffusion and kurtosis tensors, respectively. A description of the DKI analysis method can be found in our previous studies (Guo et al., 2016; Zheng et al., 2017).

The raw ASL data were also transferred to the GE Advantage Workstation 4.6 and post-processed by the ReadyView software (version 10.3.67) in the FuncTool environment. The CBF values were reacquired before the quality control step as follows. First, a high level of background suppression was used to suppress the surrounding static tissue around the pseudocontinuous labeling pulse. Second, motion correction, temporal and spatial filtering, and partial volume effect correction were performed. Subsequently, the CBF values were calculated by intensity normalization. A quantitative CBF map was generated based on the following equation:

$$\begin{array}{l} \text{CBF}=\frac{\lambda \left(1-{\text{e}}^{\frac{-{\text{t}}_{\text{sat}}}{{\text{T}}_{1\text{g}}}}\right)}{2\alpha {\text{T}}_{1\text{b}}\left(1-{\text{e}}^{\frac{\tau }{{\text{T}}_{1\text{b}}}}\right)}\frac{\Delta \text{S}}{{\text{S}}_{0}}{\text{e}}^{\frac{\omega }{{\text{T}}_{1\text{b}}}} \end{array}$$

where T_1b_ represents the T1 relaxation time of the blood, T_1g_ represents the T1 relaxation time of the GM, t_sat_ represents the duration time of the saturation pulse performed before imaging, α represents the labeling efficiency, λ represents the brain/blood partition coefficient, τ represents the labeling duration, and ω represents the post-labeling delay time. In addition, CBF analysis has been described in previous studies (Wu et al., 2014; Zhao et al., 2016).

**Figure 1:**
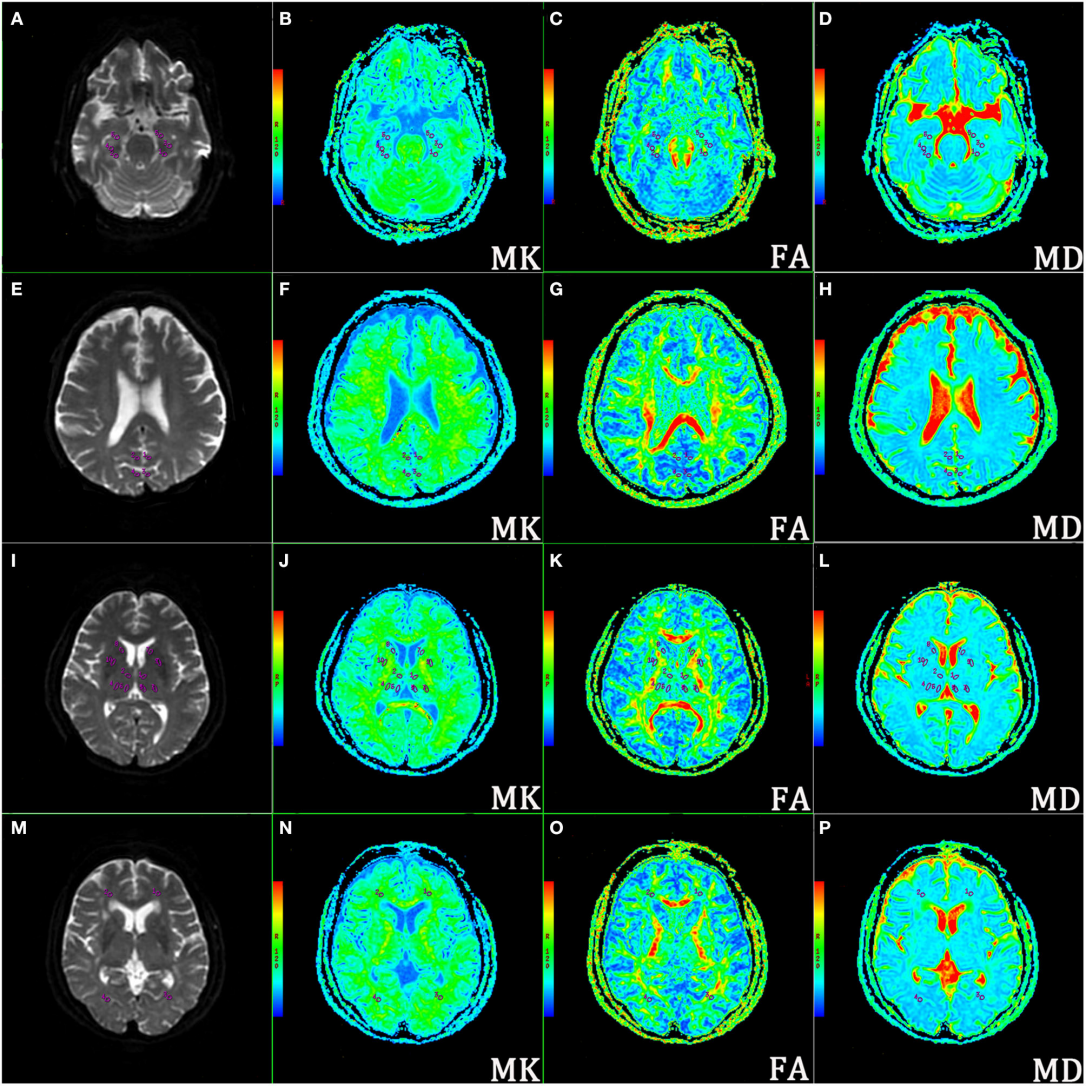
Representative locations of the regions of interest (ROIs) on corresponding axial b0 images and mean kurtosis (MK), fractional anisotropy (FA), and mean diffusivity (MD) maps. **(A–D)** ROIs of the bilateral hippocampal (Hip) subregions (head, body, and tail); **(E–H)** ROIs of bilateral posterior cingulate cortex (PCC) and precuneus (Pr); **(I–L)** ROIs of bilateral dorsal thalamus (DT) subregions (anterior nucleus, ventrolateral nucleus, and medial nucleus), lenticular nucleus (LN), and caput nuclei caudati (CNC); **(M–P)** ROIs of bilateral white matter of the occipital lobe (OLWM) and white matter of the frontal lobe (FLWM).

Regions of interest (ROIs) were carefully delineated and analyzed by two independently experienced neuroradiologists who were blinded to the status of the subjects with B0 images as references. Each ROI was selected on the maximum level of the structures that were best demonstrated according to each corresponding parametric map. When the ROIs were placed, we also tried to avoid major vascular structures, cerebrospinal fluids, and interhemispheric regions. To ensure that the same locations were extracted from each subject, the ROIs (the ROI areas ranged from 18 to 22 mm^2^) in the brain were mainly obtained from 24 regions using mirror symmetry tools (after drawing an ROI in the left hemisphere, it is copied and then left-to-right flipped across the brain midline to obtain the same shape and size of ROI) derived from FuncTool software, as shown in Figures 1A–P for the DKI maps and Supplementary Figures 1A–D for the ASL maps. These regions included the bilateral Hip subregions [head; Hip (h), body; Hip (b) and tail; Hip (t)], PCC, Pr, and DT subregions [anterior nucleus: DT (a), ventrolateral nucleus: DT (vl), and medial nucleus (m)], lenticular nucleus (LN), caput nuclei caudati (CNC), FLWM, and OLWM. The values of MK, FA, MD, and CBF were calculated and averaged across three replicates by two senior radiologists to correct and reduce offset errors. After that, an intraclass correlation coefficient (ICC) analysis was performed to further assess the consistency of MK, FA, MD, and CBF obtained from the measurements taken by two neuroradiologists (Landis and Koch, 1977). Usually, ICC values >0.75 are regarded as a good correlation.

### Statistical Analysis

Statistical analyses were performed using Statistical Package for Social Science version 25.0 (SPSS Inc., Chicago, III, USA) and GraphPad Prism software 8.3.0 (https://www.graphpad.com/scientific-software/prism). Sex distributions were analyzed using the chi-squared test among the three groups. One-way ANOVA was used to assess age, education, imaging metrics, and neuropsychological test results among patients with SCD plus, patients with aMCI, and ECs. ANOVA with false discovery rate (FDR) correction as described by the Benjamini–Hochberg method was used to compare the DKI parameters and CBF values using the R soft package (R for Windows v. 4.0.3, https://cran.r-project.org) among three groups to compare the symmetrical ROIs in the left and right cerebral hemisphere, respectively. The non-parametric Kruskal–Wallis test was used if the data were not normally distributed. Pearson's correlation analysis was used to evaluate the relationship between the measured parameters and the key neuropsychological scores for SCD plus controlled for age, sex, and the education level as nuisance covariates. The measured neuroimaging parameters were determined by receiver operating characteristic (ROC) curve analysis to evaluate the diagnostic accuracy. To determine the predictive accuracy of the parameters in discriminating patients with SCD plus from patients with aMCI or ECs, the area under the ROC curve (AUC) was calculated to assess the diagnostic ability of the imaging metrics, both alone and in combination. Combined AUC values were obtained by binary logistic regression. All data were expressed as means ± SD, and the values of *p* < 0.05 were considered statistically significant.

## Results

### Demographic and Key Neuropsychological Assessment Scores in Participants With SCD Plus, aMCI, and ECs

The baseline demographic characteristics and key neuropsychological scores of the studied participants are presented in Table 1. No significant differences were found with respect to age, sex, and education among participants with SCD plus, aMCI, and ECs. The AVLT, MMSE, and MoCA scores decreased gradually among the three groups. Only the MMSE scores and MoCA scores were lower in participants with aMCI than in ECs. Only the AVLT-DR scores between patients with SCD plus and ECs were significantly different. The SCD plus group had significantly lower AVLT-DR and AVLT-recognition scores than the aMCI group. Additionally, all subgroups of the AVLT scores, including AVLT-immediate recall scores, AVLT-DR scores, and AVLT-recognition scores, were significantly lower in patients with aMCI than in ECs.

**Table 1:** Demographic information and key neuropsychological tests for participants with SCD plus, participants with aMCI, and ECs.

**Characteristics**	**Groups**			**χ^**2**^/F/t**	***P-*value**	**Multiple comparison** ***p*****-values**		
	***ECs (*n* = 33)***	***SCD plus (*n* = 27)***	***aMCI (*n* = 31)***			***SCD plus vs. ECs***	***SCD plus vs. aMCI***	***aMCI vs. ECs***
Age (years)	67.061 ± 6.067	68.074 ± 7.014	68.645 ± 6.879	0.227	0.826	0.929	0.986	0.642
Sex (M/F)	13/20	12/15	12/19	0.615	0.735	0.803	0.599	0.439
Education (years)	10.061 ± 1.619	9.852 ± 1.747	9.907 ± 1.578	0.632	0.557	0.951	0.248	0.055
AVLT-immediate recall (score)	8.727 ± 1.292	8.111 ± 1.281	7.335 ± 1.119	5.035	<0.01	0.069	0.053	<0.01
AVLT-delayed recall (score)	8.970 ± 1.185	7.556 ± 0.934	5.387 ± 1.066	11.396	<0.001	<0.05	<0.01	<0.001
AVLT-recognition (score)	10.061 ± 1.166	9.370 ± 1.214	8.419 ± 1.317	6.653	<0.01	0.081	<0.01	<0.05
MMSE (score)	27.879 ± 1.783	26.856 ± 2.135	25.386 ± 1.317	3.722	<0.05	0.168	0.056	<0.05
MoCA (score)	26.324 ± 2.205	25.763 ± 2.573	22.365 ± 3.216	5.808	<0.01	0.159	0.052	<0.01

### Changes in the DKI Parameters and CBF in Participants With SCD Plus, aMCI, and ECs

**Figure 2:**
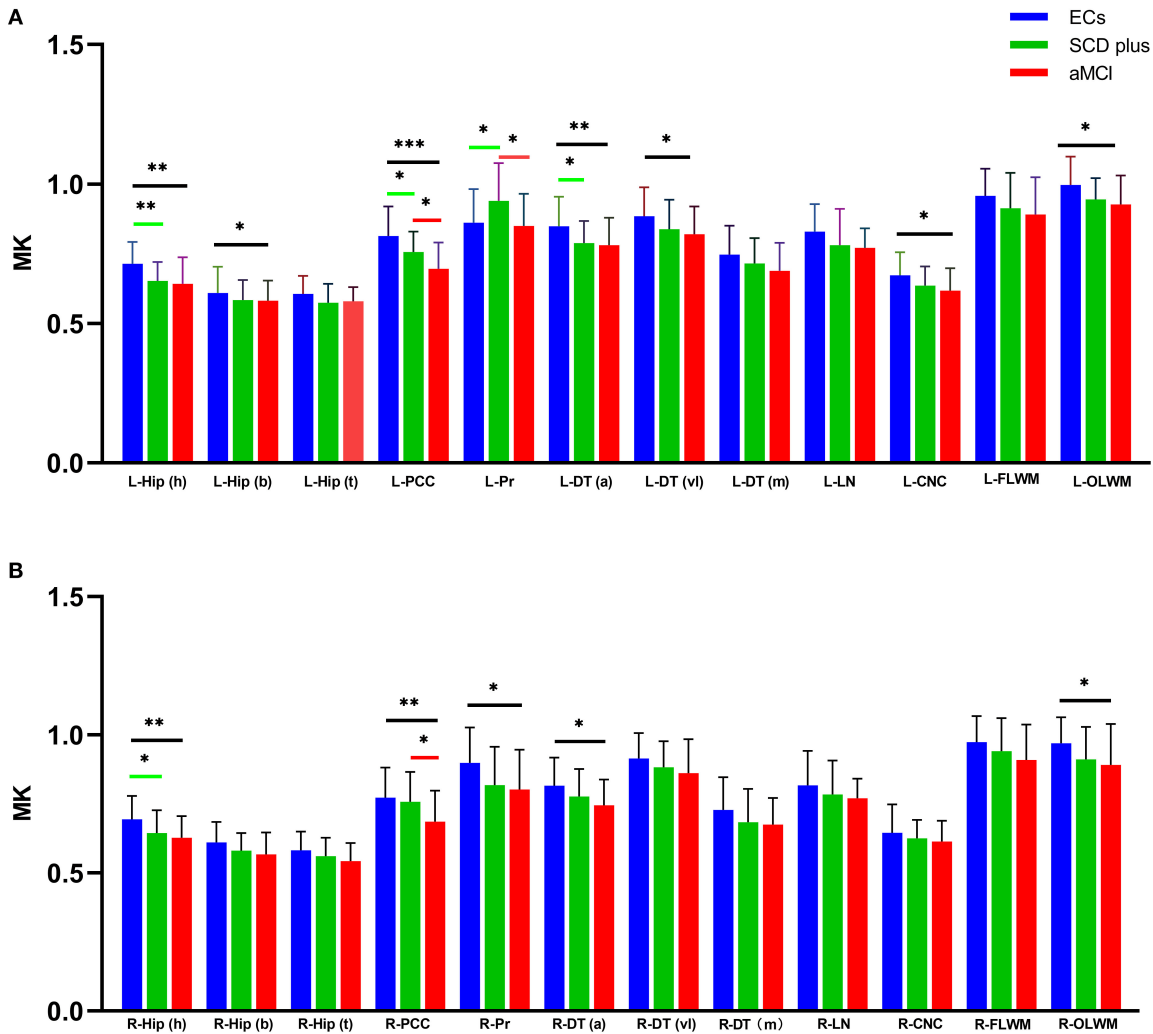
Groups differences in all measured MK values from the left **(A)** and right **(B)** ROIs. ^*^p < 0.05, ^**^p < 0.01, and ^***^p < 0.001. [false discovery rate (FDR)-corrected].

**Figure 3:**
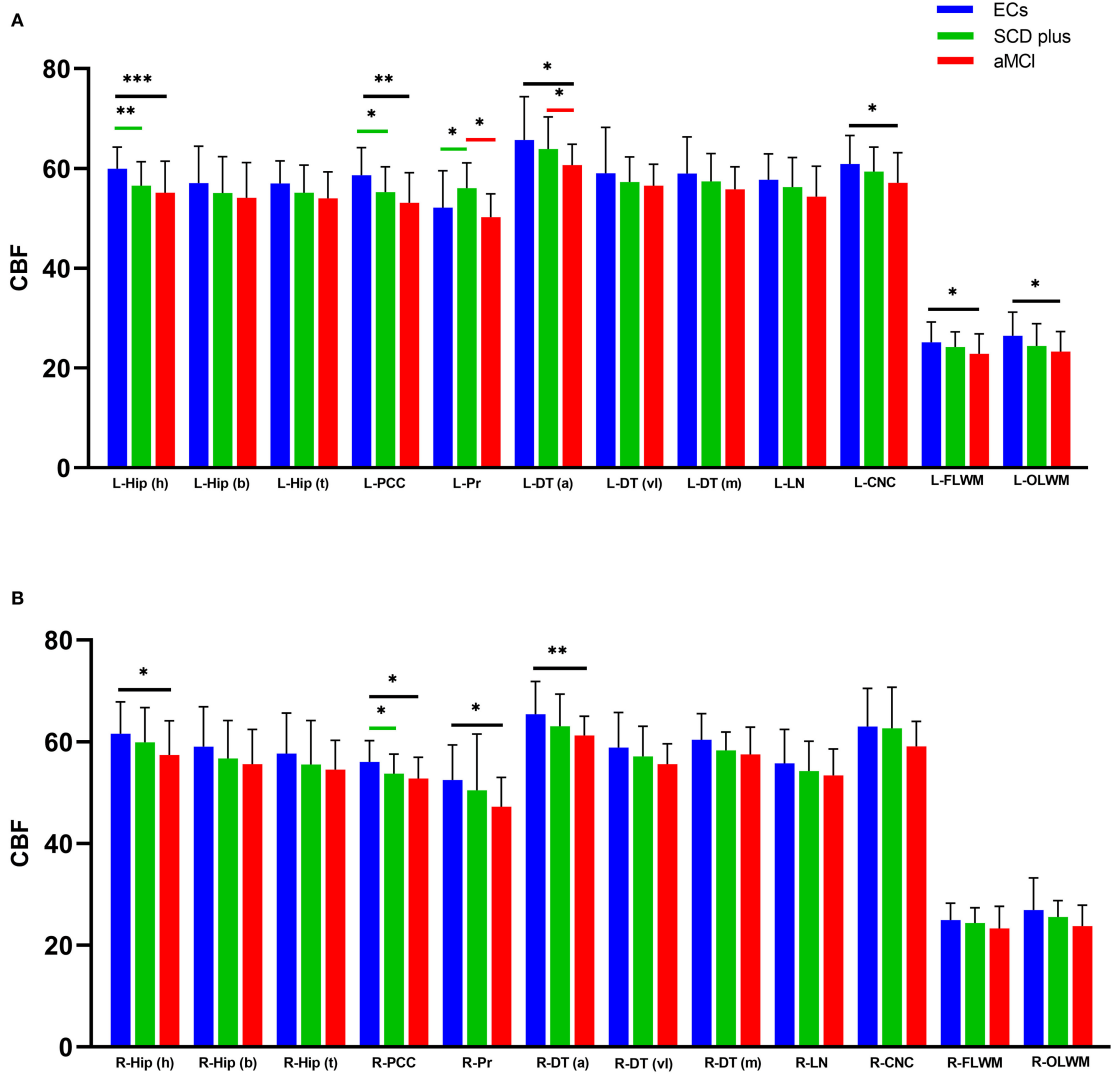
Groups differences in all the measured cerebral blood flow (CBF unit: ml/100g/min) values from the left **(A)** and right **(B)** ROIs. ^*^p < 0.05, ^**^p < 0.01, and ^***^p < 0.001. (FDR-corrected).

Mean kurtosis, FA, MD, and CBF values were acquired in the bilateral Hip (h), Hip (b), Hip (t), PCC, Pr, DT (a), DT (vl), DT (m), LN, CNC, FLWM, and OLWM among the three groups (Supplementary Raw Data). Supplementary Table 1 showed the ICC analysis of DKI and 3D-ASL parameters in the left and right ROIs in individuals with SCD plus, aMCI, and ECs. The ICC values showed that all measurements were > 0.75 and were regarded as relatively reliable among the three groups. Therefore, the levels of MK, FA, MD, and CBF were used for subsequent statistical analysis. Figures 2A,B, 3A,B showed the differences in all the measured MK and CBF values from the left and right ROIs among the three groups, respectively. Supplementary Figures 2A,B, 3A,B showed the differences in the FA and MD values from the left and right ROIs among the three groups, respectively. Supplementary Tables 2–4 showed the differences in the MK, FA, MD, and CBF values from all the ROIs among the three groups.

Compared with the ECs, participants with SCD plus had significantly lower MK values in the bilateral Hip (h), left PCC, left DT (a), and left OLWM. Decreased CBF values were also found in the left Hip (h) and bilateral PCC. However, the MK and CBF values were increased in the left Pr. The FA value was decreased only in the left OLWM. No significant MD changes were found in any ROI between the two groups.

Compared with ECs, participants with aMCI had a lower level of MK in the bilateral Hip (h), left Hip (b), bilateral PCC, bilateral DT (a), left DT (vl), bilateral OLWM, and left CNC. Similarly, the level of CBF was decreased in the bilateral Hip (h), bilateral PCC, bilateral DT (a), left OLWM, left CNC, and left FLWM. The FA levels were also decreased in the bilateral PCC, left Pr, bilateral OLWM, and bilateral FLWM. Additionally, the MD values were elevated in the left Hip (h), left PCC, left DT (a), and left OLWM. No significant differences in the MD values were found in any right ROIs between the two groups.

Compared with participants with SCD plus, patients with aMCI had lower MK levels in the bilateral PCC and left Pr. Decreased FA values in the right OLWM along with the CBF levels in the left Pr and the left DT (a) were also observed. No significant differences were found in the MD values in any ROIs, the FA values in the left ROIs, or the CBF levels in the right ROIs between the two groups.

### Diagnostic Performance of the MK and CBF Values and Their Correlations With Key Neuropsychological Scores From Patients With SCD Plus

**Figure 4:**
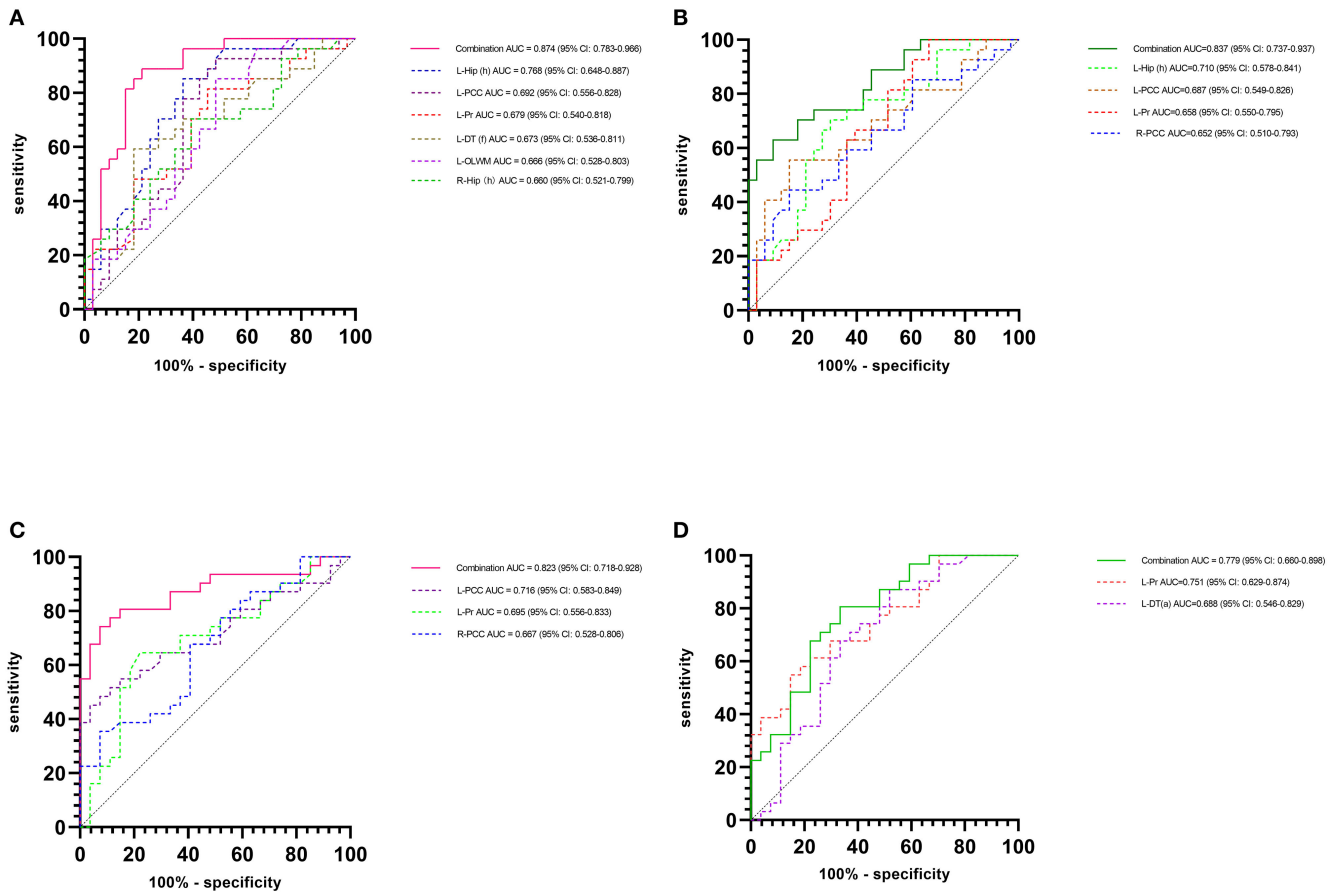
Receiver operating characteristic (ROC) curves of the discriminatory power between subjective cognitive decline (SCD) plus and elderly controls (ECs)/amnestic mild cognitive impairment (aMCI) individuals. The combined area under the ROC curve (AUC) in discriminating SCD plus from ECs obtained with MK **(A)** and CBF values **(B)**. Combined AUC in discriminating participants with SCD plus from participants with aMCI obtained with MK values **(C)** and CBF values **(D)**.

**Figure 5:**
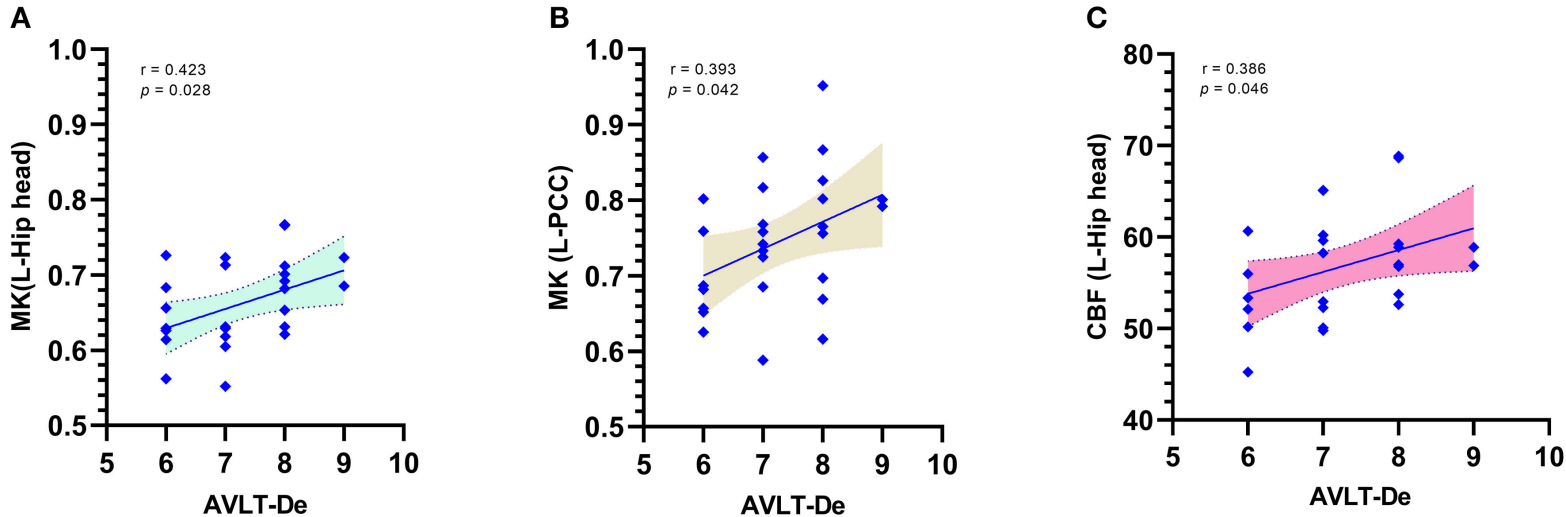
Correlations between the MK/CBF values and Auditory Verbal Learning Test-delayed recall (AVLT-DR) scores for SCD plus after adjusting for the effects of age, sex, and the education level. Positive correlations between the AVLT-DR scores and MK levels in the L-Hip head **(A)** and L-PCC **(B)**. Positive correlations between the AVLT-DR scores and CBF in the L-Hip head **(C)**. AVLT-DR, Auditory Verbal Learning Test-delayed recall.

The above results showed that the changes in the DKI and the ASL parameters in patients with SCD plus and patients with aMCI are multiregional, particularly the MK and CBF values. Thus, combined AUC values with multiple subregions, rather than a single AUC, might better distinguish among the groups, provide a better cumulative accuracy, and supply more useful clinical information. Therefore, we mainly evaluated the changes in MK or CBF, which could serve as a good imaging biomarker for a more accurate discrimination of patients with SCD plus from ECs or patients with aMCI using the ROC curve analysis. When we used a single AUC, we found that the MK values in the left Hip (h) had the best group discriminability, with an AUC of 0.768 (95% CI: 0.648–0.887) for the “SCD plus vs. ECs” comparison, while the CBF value in the left Pr had the best group discriminability, with an AUC of 0.751 (95% CI: 0.629–0.874) for the “SCD plus vs. aMCI” comparison. However, based on the combined AUC analysis method, the combined AUC (Figure 4A) in discriminating patients with SCD plus from ECs obtained using the MK levels of the bilateral Hip (h), left PCC, left Pr, left DT (a), and left OLWM increased to 0.874 (95% CI: 0.783–0.966), while the combined AUC (Figure 4B) in discriminating the two groups obtained using the CBF values in the left Hip (h), left PCC, left Pr, and right PCC increased to 0.837 (95% CI: 0.737–0.937). Similarly, the combined AUC (Figure 4C) in distinguishing patients with SCD plus from patients with aMCI obtained using the MK values in the left PCC, left Pr, and right PCC increased to 0.823 (95% CI: 0.718–0.928). The combined AUC (Figure 4D) in distinguishing patients with SCD plus from patients with aMCI obtained using the CBF values in the left Pr and left DT (a) increased to 0.779 (95% CI: 0.660–0.898). Furthermore, correlation analyses between key cognitive scores and MK/CBF values were performed for patients with SCD plus after adjusting for age, sex, and education levels. Positive correlations were found between the AVLT-DR score and the MK values in the left Hip (h) (*r* = 0.423; *p* = 0.028) and in the left PCC (*r* = 0.393; *p* = 0.042) (Figures 5A,B, respectively). However, the CBF level was positively correlated with the AVLT-DR score (*r* = 0.386; *p* = 0.046) only in the left Hip (h) (Figure 5C). No significant correlations were found between the remaining DKI or ASL parameters in these regions and key cognitive scores in patients with SCD plus.

## Discussion

Subjective cognitive decline is known to be pervasive in elderly individuals older than 65 years (~25–56%), and approximately half of them remain cognitively stable (Jonker et al., 2000). Although SCD is associated with a risk of dementia, the rate of development to MCI/AD is relatively low due to its heterogeneity (Perrotin et al., 2017). To the best of our knowledge, this is the first study to investigate brain tissue microstructural and perfusion changes, including those in bilateral Hip and DT subfields, in patients with SCD plus. In this study, we used the DKI and 3D-ASL techniques, which combine features from different regional distributions of imaging metrics, combine output data from multiple voxels, and generate a wealth of information. Thus, we could detect the CBF and microstructural abnormality distribution in different brain regions and obtain highly accurate diagnostic information using these practical techniques. First, our main findings showed that SCD plus group exhibits microstructural and perfusion alterations in multiple encephalic regions, particularly in the Hip (h) and PCC. The aMCI group exhibited more significant abnormalities in MK and CBF values than ECs and patients with SCD plus in multiple regions. These results indicated that patients with SCD plus might share a similar trend of microstructural and perfusion changes with individuals with aMCI (Sánchez-Benavides et al., 2018; Hao et al., 2020). Second, MK seems to contribute more significantly than CBF to the diagnostic performance in patients with SCD plus when combined AUC values are used. Moreover, the changes in MK values in the left Hip (h) and left PCC of the SCD plus group were positively correlated with AVLT-DR scores, whereas the CBF value in only the left Hip (h) was positively correlated with the AVLT-DR scores.

In a previous series of studies, patients with aMCI and patients with AD demonstrated aberrant microstructures according to DKI (Struyfs et al., 2015; Chen et al., 2017; Cheng et al., 2018; Song et al., 2019) or CBF changes according to ASL (Yoshiura et al., 2009; Haller et al., 2016; Fällmar et al., 2017; Riederer et al., 2018) in many brain regions. In this study, we also observed CBF and microstructural abnormalities in the Hip, PCC, Pr, OLWM, DT, FLWM, and CNC regions in patients with aMCI, a finding that agrees with findings from the previous study describing the association of changes in these regions with the likelihood of AD. Two studies investigated SCD-related ASL findings, and the results were inconsistent. One study showed that, compared with ECs, patients with SCD had decreased CBF in the medial orbitofrontal cortex and increased CBF in the right putamen (Hays et al., 2018), while the other study found no discrepancy in the total CBF between patients with SCD and ECs (de Eulate et al., 2017). Our study showed decreased CBF values in the left Hip (h), bilateral PCC, and increased CBF in the left Pr. The inconsistent results were compared with our results due to the inclusion criteria being different (our study population is SCD plus, while the others mainly chose patients with SCD). Another possible reason was the different scanning protocols and parameters used, or different post-processing analysis method performed, which might also make the results different. Additionally, different sample sizes can also lead to different results. However, our main purpose was to explore brain microstructural changes and CBF alterations in patients with SCD plus, as well as their relationships with key neurocognitive scores. Additionally, we compared the advantages of MK and CBF in distinguishing patients with SCD plus from ECs or patients with aMCI.

Alzheimer's disease is a diffuse neurodegenerative disease involving many brain regions, including GM and WM (Cappa et al., 2001). As a relatively new MRI technique, DKI is sufficiently sensitive for detecting brain microstructural changes in GM and WM simultaneously, similar to ASL. In this study, MK and CBF values showed more robust and significant changes than FA or MD values among the three groups. MK, the most commonly used DKI parameter, is related to the complexity of the microstructure and is considered a complex micro index. Theoretically, non-Gaussian water molecule diffusion in the brain tissue microarchitecture is restricted by many factors, such as the cell membrane, axons, and myelin (Hansen, 2019). Degeneration and atrophy of nerve cells lead to a decline in the complexity of the brain microstructure during the course of AD, leading to a decrease in the values of MK in turn. The decline in CBF values may reflect neuronal dysfunction and synaptic failure (Hays et al., 2016). A controversy remains regarding whether the CBF values measured by ASL show hypoperfusion or hyperperfusion patterns in SCD or MCI, and the hypoperfusion/hyperperfusion areas may involve different brain regions simultaneously (Alexopoulos et al., 2012; Bron et al., 2014; Leeuwis et al., 2017; Chau et al., 2020; Kim et al., 2020). Compared with those of ECs, we found decreased MK values in the bilateral Hip (h), left PCC, left DT (a), and left OLWM and increased MK values in the left Pr in participants with SCD plus. Significantly, lower CBF values in the left Hip (h) and bilateral PCC and higher CBF values in the left Pr were also observed in participants with SCD plus. Both evaluations revealed changes in most regions in the left cerebral hemisphere, particularly in vulnerable areas such as the Hip, PCC, and Pr. The affected brain regions (left cerebral hemisphere) more often tend to involve functional asymmetry and cerebral laterality (Agarwal et al., 2016). The pronounced asymmetry between the left and right brain hemispheres is a hallmark of humans because the left cerebral hemisphere is more involved in language skills, memory function, and logic problems (Keller et al., 2018; Corballis, 2019). Furthermore, the AUC in discriminating patients with SCD plus from ECs obtained using the combined MK values was 0.874, which was higher than the combined AUC of 0.837 obtained using the CBF values. The combined AUC in discriminating patients with SCD plus from patients with aMCI obtained using the MK levels was 0.823, which was also higher than the combined AUC of 0.779 obtained with the CBF levels. These results showed that the combined AUC obtained with MK values made more significant contributions to the classification between participants with SCD plus and ECs/participants with aMCI than those calculated using the CBF values. Several possible reasons may explain this observation. The pathological changes in AD are accompanied by significantly reduced numbers of neurons as nerve cells undergo degeneration and atrophy (Morris and Price, 2001). The course of AD is associated with disruptions to mitochondrial function, decreased apoptosis, and decreased neuronal shrinkage (Leuner et al., 2007). Previous articles have suggested that mitochondrial dysfunction plays a key role in the early stage of AD (Cadonic et al., 2016). Several reports have indicated that the mitochondrial function is disrupted in patients with MCI and patients with AD (Delbarba et al., 2016; Mastroeni et al., 2017; Terada et al., 2020). Mitochondrial dysfunction, in turn, accelerates the production of reactive oxygen species, further contributing to oxidative stress and damage to cell membrane lipids, intracellular proteins, and DNA (Butterfield and Boyd-Kimball, 2020). The accumulation of β-amyloid (Aβ) peptides decreases ATP production, impairs the mitochondrial membrane potential, and exacerbates oxidative stress (Swomley and Butterfield, 2015). Moreover, reactive oxygen species generated from mitochondrial dysfunction can drive amyloidogenesis and tau phosphorylation, thus generating a cycle that decreases energy utilization for normal cellular function and drives the pathology and progression of nerve cell death in AD (Lejri et al., 2019). Due to neuron cell body death and loss and neuronal atrophy and apoptosis, the complexity of the brain tissue decreases, resulting in lowered MK as part of this process. MK is also a good probe for the presence of cell microstructural impairment and is sensitive in detecting the underlying Aβ-induced pathology (De Santis et al., 2011). Therefore, the slight modification in MK values found in this study could be an early reflection of mitochondrial energy metabolism dysfunction in conditions with a fairly high risk of the very early stage of AD. The decrease in the CBF value can also be attributed to the inhibited activity or dysfunction of neurons or neural synaptic loss, as well as the changes in neural activity due to the progression of AD pathology (Hays et al., 2016). As described above, the mitochondria, which convert oxygen and a metabolic product of glucose into ATP to generate energy, are the powerhouses of cells, including neurons; mitochondrial dysfunction in nerve cells will cause them to function improperly. As the activity of the neuron changes, the demand for oxygen decreases (mainly in the mitochondria), and the demand for energy (ATP production) decreases further, moderating the CBF (Ogoh and Ainslie, 2009). Therefore, mitochondrial dysfunction may precede changes in CBF. Our results also suggested that changes in MK are observed slightly before those in CBF. Measurement of the changes in MK seems to be a promising method for evaluating participants at risk for dementia because they may reflect early and impaired energetic metabolism in the brain, a factor involved in the pathogenesis of AD. Of course, although we excluded vascular factors (high blood pressure, diabetes, and WM hyperintensity lesion burden), diagnosis biases, heterogeneity factors, and/or individual variability of disease expression with SCD plus should still be considered (Jessen et al., 2014). Additionally, limitations related to the ASL techniques, including their sensitivity to transit time effects and relatively low spatial and space resolution, must also be considered.

Our results are consistent with the findings of a study reporting that GM hypometabolism and microstructural abnormalities are the core characteristics of aMCI/AD (Gong et al., 2017). The focal areas in patients with SCD plus and patients with aMCI are mainly in the limbic system, including the Hip, PCC, and Pr, which are predominantly related to neuropsychological activities such as learning, memory, the generation of emotional responses, and behavior (Zhang et al., 2014). Moreover, the ROI analysis revealed significantly altered values of MK/CBF in the DT and basal ganglia of participants with SCD plus that also play an important role in attention and working memory (Cho et al., 2014; de Bourbon-Teles et al., 2014). Compared with the ECs, the individuals in the SCD plus group showed the most obvious abnormalities in MK/CBF values in the Hip (h). Previous studies have shown that the Hip (h) plays a key role in memory performance and is the most sensitive and vulnerable region to memory impairment among the Hip subregions (Ouchi et al., 1998; Yakushev et al., 2010). It may also be the earliest region susceptible to pathological changes in patients with aMCI/AD (Gordon et al., 2013; Luo et al., 2014). The detection of reduced MK/CBF in the left Hip is also consistent with the previous results of DKI/ASL perfusion studies (Dai et al., 2009; Binnewijzend et al., 2013; Gong et al., 2017; Cheng et al., 2018; Song et al., 2019). As a limbic cortical region, the PCC is affected relatively early in the pathological progression to AD. The decreased MK/CBF levels may be tightly associated with the derangement of neurometabolism and neuronal/axonal loss (Thomas et al., 2019). The exploration of other ROIs, such as in the left DT (a) and left OLWM, could also aid in improving predictions. However, decreased or increased MK/CBF can coexist in the early stages of the neurodegenerative process. Our results showed that compared with ECs, patients with SCD plus had higher values of MK/CBF only in the left Pr. The hyperperfusion patterns are thought to reflect compensatory mechanisms for the neuronal damage that occurs early in the disease process to counterbalance cognitive decline (Sierra-Marcos, 2017). We speculate that the beneficial effects of elevated MK/CBF values on cognition may be destroyed in patients with SCD plus because decreased MK/CBF values in most brain regions and only higher levels of MK/CBF in the left Pr cannot support normal cognitive function within regions associated with normal aging and AD risk. Alternatively, the results may reflect changes in the brain microvasculature due to increased neurocerebrovascular reactivity without any significant gains in the cognitive performance (Miki et al., 2009; Sam et al., 2016). Therefore, the present observations combining MK and CBF measures clearly support this view.

Consistent with most previous DTI studies, we observed decreased FA and increased MD values between the EC and aMCI groups. However, decreased FA values were found only in the left OLWM in the EC and SCD plus groups. Additionally, abnormalities in FA values were found only in the right OLWM between participants with SCD plus and participants with aMCI. FA represents directional variation in water molecule diffusion, whereas MD reflects the average diffusivity. The observed decline in FA and increase in MD values parallel the axonal loss and myelin damage that restrict the random motion of water molecules along the nerve fiber tracts. Consistent with a previous study, our results demonstrate that FA is preferable to MD for evaluating WM (OLWM and FLWM) (Medina et al., 2006; Chen et al., 2009). Additionally, FA is more advantageous than MD in detecting early-stage AD (Yang D. W. et al., 2012). In general, among the three DKI parameters, MK is the most sensitive metric for capturing GM and WM microstructural abnormalities in patients with SCD plus and aMCI (Struyfs et al., 2015).

Furthermore, significant correlations between AVLT-DR scores and MK values in the left Hip (h) and PCC were observed in patients with SCD plus. A correlation only between the AVLT-DR scores and CBF values was observed in the left Hip. We assumed that the decreased MK/CBF in the left Hip (h) may be crucial for progressive memory impairment. Our correlation results also support the possibility that changes in MK are the strongest predictor of cognitive impairment scores, particularly in the very early stage of AD.

## Conclusion

Several limitations of this study should be noted. First, as a cross-sectional study, few SCD plus patients were included. The sample size should be increased, and a longitudinal study of this study population is needed to identify early neuroimaging biomarkers for the prediction of AD. Second, patients with AD were not included in our study; therefore, the continuous spectrum of AD could not be fully explored. Further research should be designed with more participants to assess the distinction among SCD plus converters, SCD plus non-converters, and probable AD. Third, the inclusion criteria for SCD plus were based on the number of SCD plus features met; typically, patients are expected to meet more than three (Jessen et al., 2014). Although the MK level is emerging as a very useful imaging marker in AD because it may strongly relate to the presence of an underlying amyloid pathology and neurodegeneration, we did not test the statuses of APOE-ε4, tau protein, or Aβ, reflecting a limitation and lack of completeness in our study. Incorporating examinations of these objective biomarkers associated with AD pathology may be more important for detecting patients with SCD plus. Finally, multimodal imaging techniques and more advanced post-processed methods (Zhang et al., 2016; Jiskoot et al., 2019) would yield a more comprehensive understanding for elucidating the pathophysiologic mechanisms underlying SCD plus (Parker et al., 2020; Wang et al., 2020).

In summary, our results suggest that DKI and ASL can provide effective information to detect the characteristics of CBF and microstructure abnormalities in patients with SCD plus. MK may provide valuable information and function as one of the earliest potential neuroimaging biomarkers associated with AD. Changes in the MK value in the Hip (h) and PCC appear to represent an optimal, effective, and potentially useful non-invasive disease biomarker of the preclinical phase of AD.

## Data Availability Statement

The original contributions presented in the study are included in the article/Supplementary Material, further inquiries can be directed to the corresponding author/s.

## Ethics Statement

The studies involving human participants were reviewed and approved by the Ethical Committee of Second Affiliated Hospital of Shantou University Medical College (Registration No. 2017-10). The patients/participants provided their written informed consent to participate in this study.

## Author Contributions

ZY, YR, and ZC: designed the study and collected the data. YR, YW, and ZC: collected the data. XZ, QX, and ML: performed the statistical analysis. ZY and YL: conceived and designed the experiments and prepared the manuscript. All authors contributed to the article and approved the submitted version.

## Conflict of Interest

The authors declare that the research was conducted in the absence of any commercial or financial relationships that could be construed as a potential conflict of interest.
