# Supplementary material for: Microstructural and Cerebral Blood Flow Abnormalities in Subjective Cognitive Decline Plus: Diffusional Kurtosis Imaging and Three-Dimensional Arterial Spin Labeling Study
Source: Front Aging Neurosci. 2021 Feb 1;13:625843. doi: 10.3389/fnagi.2021.625843 (PMC7882515; doi:10.3389/fnagi.2021.625843)
Supplement: Supplementary file 1 [file Data_Sheet_1.zip › Data Sheet 1/Supplementary materials/Supplementary Material (Figure 1-3 and Table 1-4).docx]

Supplementary Material

**Supplementary Figure 1.** Representative locations of the volumes of interest (VOIs) on corresponding axial CBF maps. A: ROIs of bilateral Hip subregions (head, body and tail); B: ROIs of bilateral PCC and Pr; C: ROIs of bilateral DT subregions (anterior nucleus, ventrolateral nucleus, medial nucleus), LN, and CNC; D: ROIs of bilateral OLWM and FLWM.


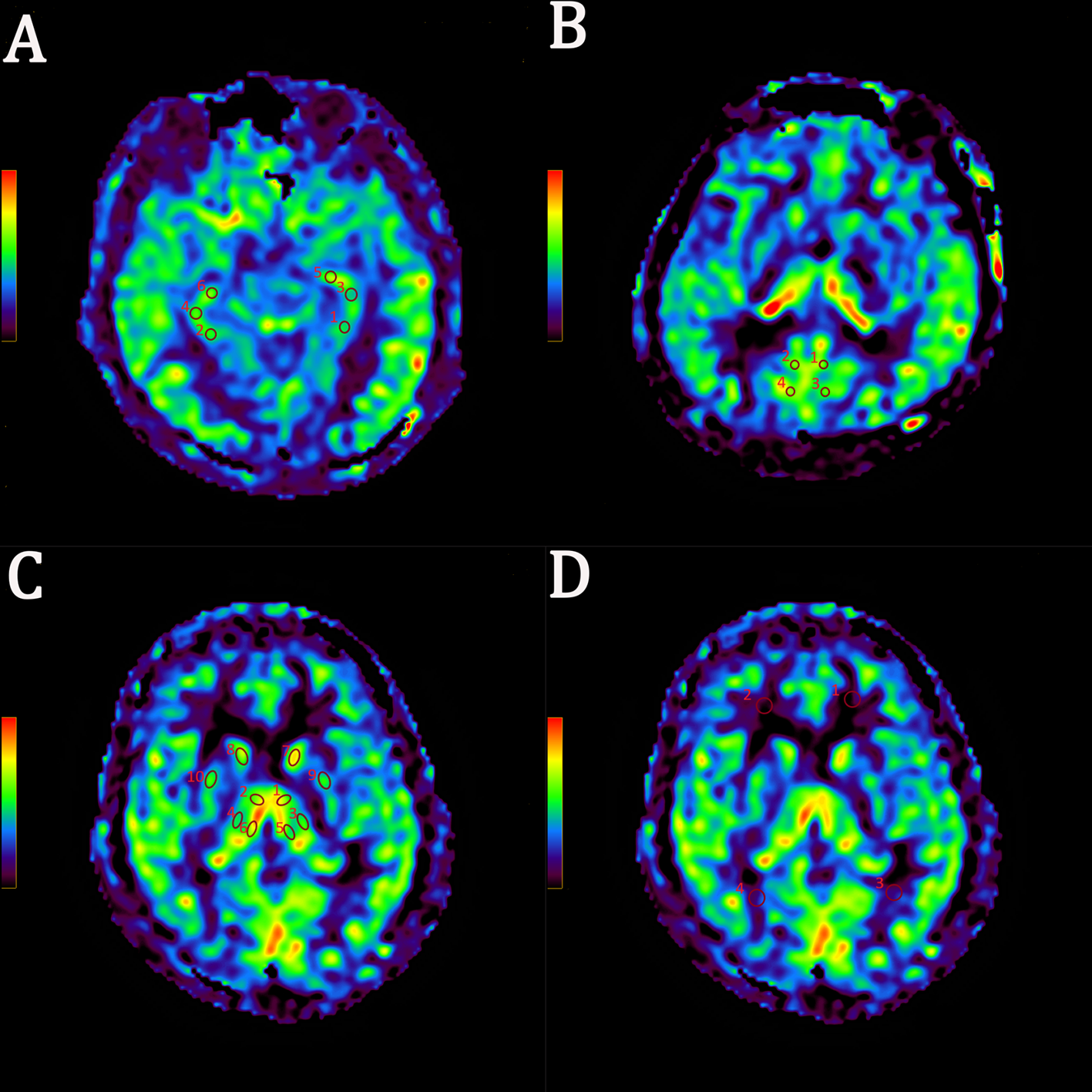


**Supplementary Figure 2.** Groups differences in all the measured FA values from the left (A) and right (B) ROIs. *p＜0.05, **p＜0.01 (FDR-corrected).


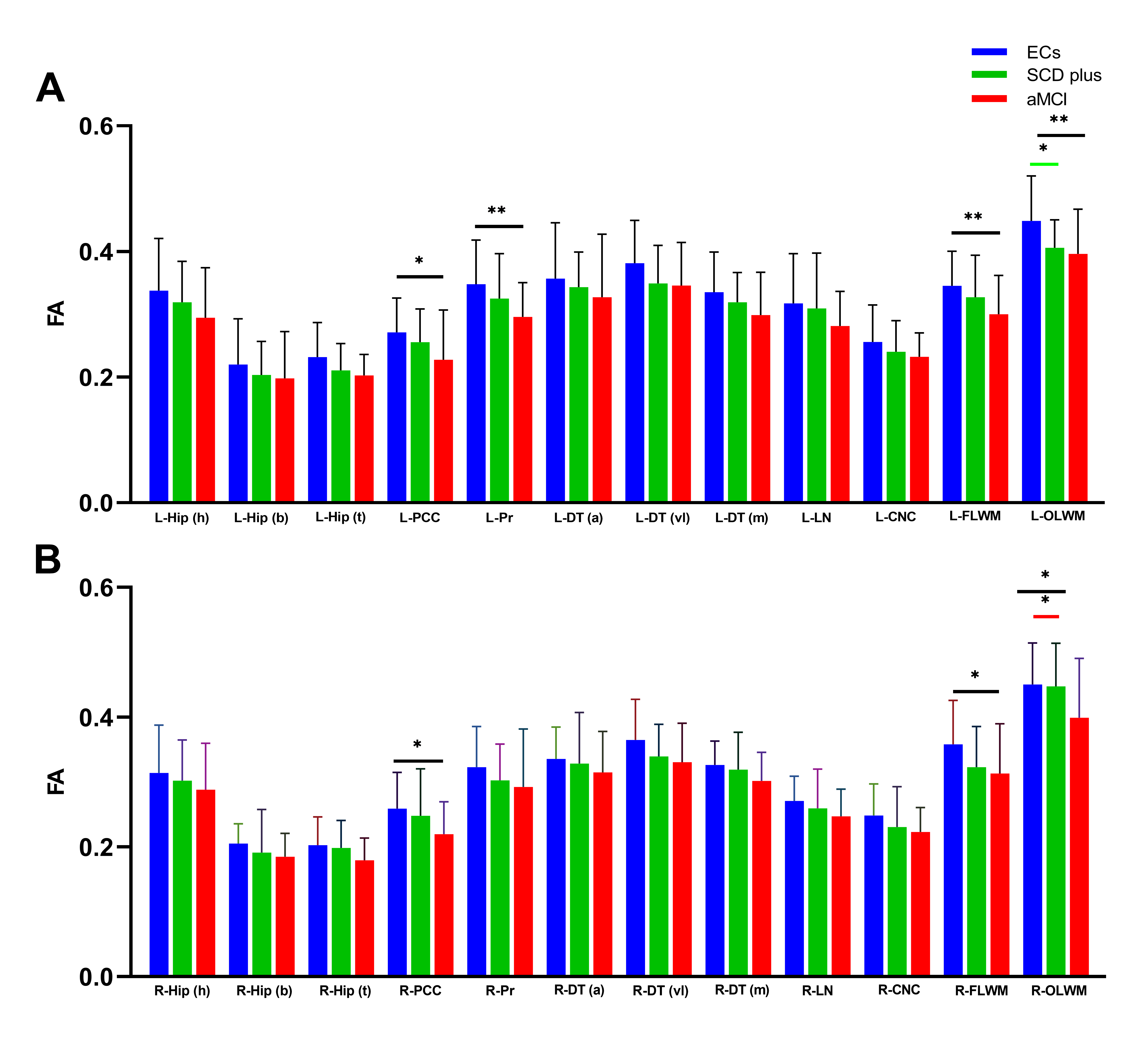


**Supplementary Figure 3.** Groups differences in all the measured MD values from the left (**A**) and right (**B**) ROIs. ^*^*p*＜0.05, ^**^*p*＜0.01 (FDR-corrected).


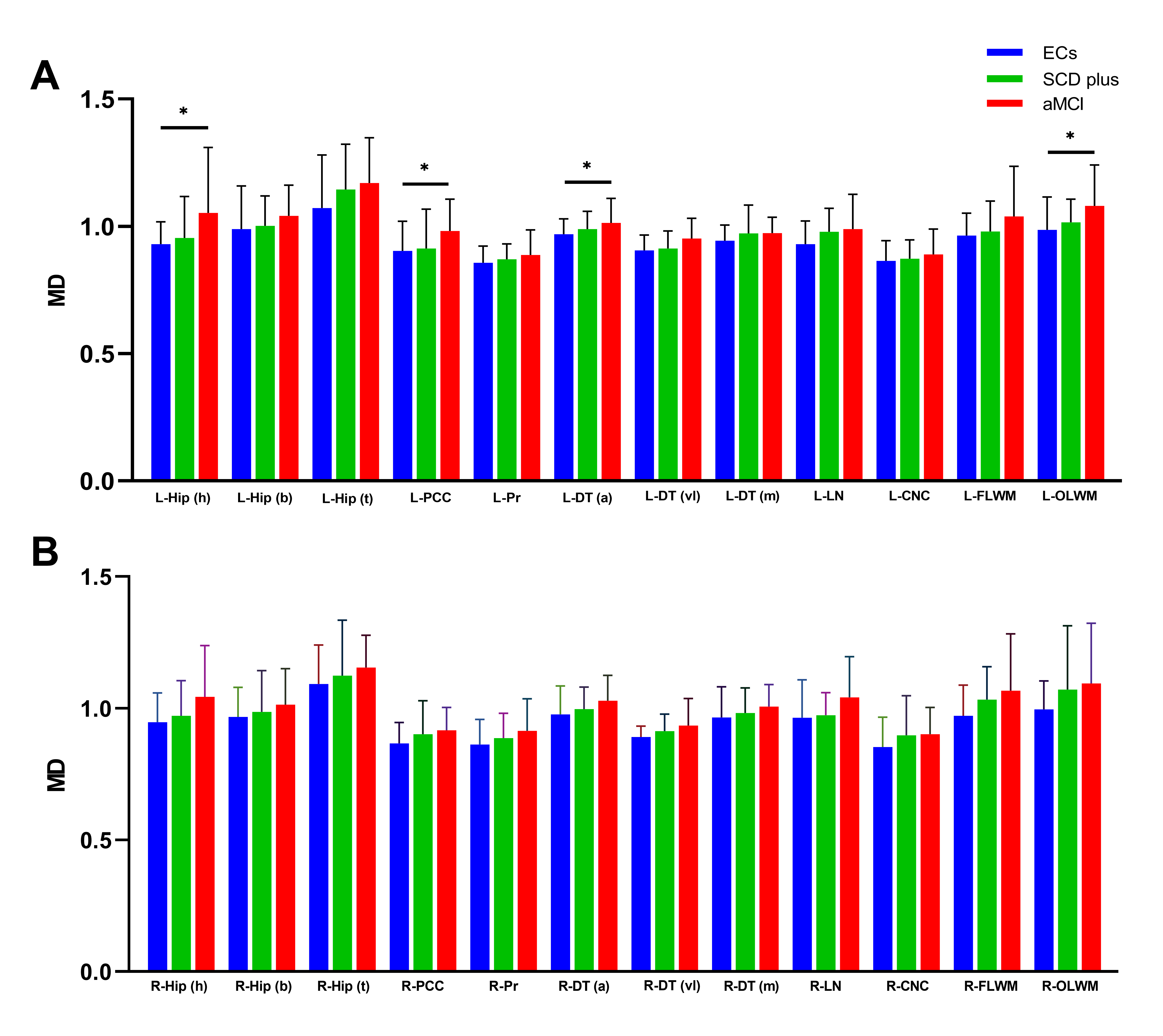


**Supplementary TABLE 1.** Intraclass correlation coefficients (ICCs) analysis of DKI and 3D-ASL parameters in the left and right ROIs in individuals with ECs, SCD plus and aMCI.

| **ROIs** | **Parameters** | ***ECs (ICCs)***  ***Left right*** | ***SCD plus (ICCs)***  ***Left right*** | ***aMCI( ICCs)***  ***Left right*** |
| --- | --- | --- | --- | --- |
| Hip (h) | MK  FA  MD  CBF | 0.821 0.834  0.872 0.836  0.911 0.924  0.769 0.782 | 0.803 0.795  0.806 0.822  0.907 0.855  0.766 0.790 | 0.819 0.883  0.873 0.806  0.843 0.815  0.818 0.825 |
| Hip (b) | MK  FA  MD  CBF | 0.789 0.885  0.842 0.885  0.820 0.832  0.819 0.823 | 0.839 0.867  0.782 0.855  0.853 0.903  0.796 0.785 | 0.861 0.826  0.906 0.851  0.918 0.893  0.772 0.760 |
| Hip (t) | MK  FA  MD  CBF | 0.908 0.905  0.797 0.850  0.909 0.881  0.781 0.767 | 0.874 0.831  0.800 0.811  0.793 0.801  0.797 0.805 | 0.851 0.788  0.866 0.768  0.841 0.827  0.816 0.778 |
| PCC | MK  FA  MD  CBF | 0.813 0.867  0.757 0.781  0.829 0.826  0.765 0.771 | 0.790 0.825  0.800 0.815  0.853 0.914  0.835 0.806 | 0.785 0.833  0.905 0.881  0.840 0.854  0.776 0.781 |
| Pr | MK  FA  MD  CBF | 0.862 0.844  0.785 0.825  0.853 0.780  0.817 0.872 | 0.813 0.909  0.836 0.860  0.769 0.852  0.806 0.783 | 0.821 0.864  0.790 0.804  0.819 0.896  0.791 0.862 |
| DT (a) | MK  FA  MD  CBF | 0.858 0.817  0.783 0.803  0.908 0.792  0.862 0.786 | 0.865 0.900  0.867 0.881  0.862 0.811  0.772 0.822 | 0.796 0.820  0.913 0.855  0.767 0.836  0.807 0.843 |
| DT (vl) | MK  FA  MD  CBF | 0.851 0.917  0.782 0.833  0.756 0.806  0.791 0.812 | 0.842 0.951  0.885 0.804  0.893 0.802  0.862 0.877 | 0.808 0.943  0.791 0.855  0.765 0.782  0.928 0.856 |
| DT (m) | MK  FA  MD  CBF | 0.823 0.846  0.775 0.804  0.934 0.886  0.772 0.815 | 0.847 0.923  0.784 0.792  0.907 0.816  0.826 0.817 | 0.803 0.807  0.841 0.852  0.857 0.916  0.871 0.843 |
| LN | MK  FA  MD  CBF | 0.834 0.871  0.832 0.851  0.928 0.916  0.760 0.818 | 0.800 0.868  0.835 0.864  0.869 0.886  0.822 0.813 | 0.877 0.794  0.780 0.840  0.805 0.765  0.867 0.838 |
| CNC | MK  FA  MD  CBF | 0.831 0.904  0.892 0.845  0.812 0.860  0.779 0.816 | 0.806 0.836  0.886 0.795  0.875 0.808  0.819 0.836 | 0.864 0.807  0.842 0.851  0.813 0.782  0.806 0.795 |
| FLWM | MK  FA  MD  CBF | 0.851 0.819  0.792 0.857  0.862 0.877  0.774 0.825 | 0.771 0.786  0.827 0.864  0.914 0.889  0.783 0.823 | 0.803 0.822  0.816 0.797  0.856 0.762  0.814 0.796 |
| OLWM | MK  FA  MD  CBF | 0.824 0.786  0.845 0.830  0.852 0.768  0.799 0.776 | 0.877 0.783  0.784 0.815  0.813 0.786  0.815 0.813 | 0.791 0.885  0.826 0.807  0.806 0.880  0.798 0.805 |

*Abbreviations: ECs, elderly controls; SCD plus, subjective cognitive decline plus; aMCI, amnestic mild cognitive impairment; MK, mean kurtosis; FA, fractional anisotropy; MD, mean diffusivity; CBF, cerebral blood flow; Hip (h), hippocampus head; Hip (b), hippocampus body; Hip (t), hippocampus tial; PCC, posterior cingulate cortex; Pr, inferior precuneus; DT (a), anterior nucleus of the dorsal thalamus; DT (vl), ventrolateral nucleus of the dorsal thalamus; DT (m), medial nucleus of the dorsal thalamus; OLWM, white matter of the occipital lobe; FLWM, white matter of the frontal lobe; LN, lenticular nucleus; CNC, caput nuclei caudati.*

**Supplementary TABLE 2.** Group differences in DKI parameters in left ROIs of ECs, SCD plus and aMCI patients.

| **RIOs** | **DKI metrics** | **Group *SCD plus (n=27) aMCI (n=31) ECs (n=33)*** | | | **F-value ANOVA**  ***p-value*** | | ***Multiple comparison p-value***  ***SCD plus vs ECs SCD plus vs aMCI aMCI vs ECs*** | | |
| --- | --- | --- | --- | --- | --- | --- | --- | --- | --- |
| 1. Hip (h) | MK  FA  MD | 0.655 ± 0.071  0.319 ± 0.066  0.964 ± 0.133 | 0.642 ± 0.091  0.294 ± 0.079  1.053 ± 0.156 | 0.716 ± 0.075  0.338 ± 0.083  0.930 ± 0.087 | 7.545  2.506  3.931 | **<0.001**  0.087  **<0.05** | **<0.01**  0.334  0.507 | 0.563  0.309  0.123 | **<0.01**  0.115  **<0.05** |
| L-Hip (b) | MK  FA  MD | 0.583 ± 0.072  0.211 ± 0.042  1.144 ± 0.178 | 0.561 ± 0.067  0.202 ± 0.033  1.160 ± 0.184 | 0.609 ± 0.094  0.245 ± 0.055  1.111 ± 0.146 | 3.240  2.348  1.224 | **<0.05**  0.102  0.300 | 0.231  0.266  0.737 | 0.348  0.538  0.214 | **<0.05**  0.255  0.162 |
| L-Hip (t) | MK  FA  MD | 0.575 ± 0.067  0.203 ± 0.056  1.002 ± 0.117 | 0.580 ± 0.051  0.197 ± 0.064  1.041 ± 0.121 | 0.606 ± 0.065  0.220 ± 0.053  0.989 ± 0.169 | 2.348  0.944  1.224 | 0.102  0.393  0.299 | 0.167  0.537  0.822 | 0.746  0.792  0.375 | 0.111  0.405  0.356 |
| L-PCC | MK  FA  MD | 0.756 ± 0.073  0.255 ± 0.053  0.913 ± 0.155 | 0.700 ± 0.094  0.228 ± 0.069  0.982 ± 0.126 | 0.814 ± 0.106  0.271 ± 0.054 0.903 ± 0.115 | 12.713  3.853  3.153 | **<0.001**  **<0.05**  **<0.05** | **<0.05**  0.266  0.798 | **<0.05**  0.176  0.0107 | **<0.001**  **<0.05**  **<0.05** |
| L-Pr | MK  FA  MD | 0.940 ± 0.134  0.296 ± 0.054  0.871 ± 0.060 | 0.850 ± 0.115  0.325 ± 0.072  0.888 ± 0.098 | 0.862 ± 0.120  0.348 ± 0.070  0.856 ± 0.057 | 4.484  4.991  1.381 | **<0.05**  **<0.01**  0.258 | **<0.05**  0.263  0.627 | **<0.05**  0.141  0.558 | 0.684  **<0.01**  0.283 |
| L-DT (a) | MK  FA  MD | 0.788 ± 0.079  0.343 ± 0.056  0.989 ± 0.069 | 0.781 ± 0.091  0.327 ± 0.100  1.014 ± 0.097 | 0.848 ± 0.106  0.357 ± 0.088  0.969 ± 0.060 | 4.873  1.027  2.703 | **<0.01**  0.364  0.073 | **<0.05**  0.671  0.822 | 0.786  0.654  0.525 | **<0.05**  0.403  0.061 |
| L-DT (m) | MK  FA  MD | 0.715 ± 0.090  0.318 ± 0.048  0.972 ± 0.111 | 0.688 ± 0.101  0.299 ± 0.068  0.973 ± 0.063 | 0.747 ± 0.104  0.335 ± 0.064  0.944 ± 0.061 | 2.783  2.930  1.330 | 0.067  0.069  0.273 | 0.678  0.522  0.424 | 0.737  0.316  1.000 | 0.053  0.074  0.077 |
| L-DT (vl) | MK  FA  MD | 0.838 ± 0.106  0.349 ± 0.060  0.913 ± 0.069 | 0.820 ± 0.100  0.349 ± 0.059  0.952 ± 0.080 | 0.884 ± 0.103  0.381 ± 0.068  0.906 ± 0.058 | 3.336  2.800  3.918 | **<0.05**  0.066  **<0.05** | 0.140  0.067  0.660 | 0.511  0.673  0.078 | **<0.05**  0.089  **<0.05** |
| L-LN | MK  FA  MD | 0.781 ± 0.130  0.309 ± 0.063  0.979 ± 0.092 | 0.772 ± 0.069  0.282 ± 0.055  0.990 ± 0.136 | 0.829 ± 0.099  0.317 ± 0.076  0.930 ± 0.091 | 2.968  1.955  2.728 | 0.057  0.149  0.069 | 0.095  0.847  0.128 | 0.779  0.327  1.000 | 0.052  0.080  0.064 |
| L-CNC | MK  FA  MD | 0.635 ± 0.069  0.240 ± 0.049  0.873 ± 0.074 | 0.618 ± 0.080  0.232 ± 0.038  0.890 ± 0.099 | 0.673 ± 0.082  0.256 ± 0.057  0.864 ± 0.080 | 4.323  1.884  0.755 | **<0.05**  0.159  0.473 | 0.085  0.420  0.625 | 0.376  0.603  0.676 | **<0.05**  0.077  0.437 |
| L-FLWM | MK  FA  MD | 0.913 ± 0.126  0.328 ± 0.067  0.979 ± 0.120 | 0.891 ± 0.134  0.300 ± 0.062  1.034 ± 0.196 | 0.957 ± 0.098  0.345 ± 0.055  0.965 ± 0.088 | 2.514  4.424  2.425 | 0.087  **<0.05**  0.096 | 0.269  0.269  0.812 | 0.708  0.170  0.231 | 0.072  **<0.01**  0.105 |
| L-OLWM | MK  FA  MD | 0.945 ± 0.076  0.406 ± 0.045  1.016 ± 0.091 | 0.927 ± 0.104  0.396 ± 0.071  1.081 ± 0.161 | 0.997 ± 0.102  0.449 ± 0.072  0.986 ± 0.129 | 4.773  5.908  4.415 | **<0.01**  **<0.01**  **<0.05** | **<0.05**  **<0.05**  0.319 | 0.425  0.721  0.128 | **<0.05**  **<0.05**  **<0.05** |

*Abbreviations: ECs, elderly controls; SCD plus, subjective cognitive decline plus; aMCI, amnestic mild cognitive impairment; MK, mean kurtosis; FA, fractional anisotropy; MD, mean diffusivity; L-Hip (h), left hippocampus head; L-Hip (b), left hippocampus body; L-Hip (t), left hippocampus tial; L-PCC, left posterior cingulate cortex; L-Pr, left inferior precuneus; L-DT (a), left anterior nucleus of the dorsal thalamus; L-DT (vl), left ventrolateral nucleus of the dorsal thalamus; L-DT (m), left medial nucleus of the dorsal thalamus; L-OLWM, left white matter of the occipital lobe; L-FLWM, left white matter of the frontal lobe; L-LN, left lenticular nucleus; L-CNC, left caput nuclei caudati. Values are presented as the mean ± SD.*

**Supplementary TABLE 3.** Group differences in DKI parameters in right ROIs of ECs, SCD plus and aMCI patients.

| **RIOs** | **DKI metrics** | **Group**  ***SCD plus (n=27) aMCI (n=31) ECs (n=33)*** | | | **F-value ANOVA**  ***p-value*** | | ***Multiple comparison p-value***  ***SCD plus vs ECs SCD plus vs aMCI aMCI vs ECs*** | | |
| --- | --- | --- | --- | --- | --- | --- | --- | --- | --- |
| R-Hip (h) | MK  FA  MD | 0.636 ± 0.083  0.302 ± 0.063  0.971 ± 0.123 | 0.627 ± 0.079  0.288 ± 0.072  1.044 ± 0.195 | 0.694 ± 0.085  0.314 ± 0.073  0.947 ± 0.111 | 5.766  1.106  2.905 | **<0.01**  0.336  0.063 | **<0.05**  0.743  0.613 | 0.433  0.497  0.167 | **<0.01**  0.472  0.081 |
| R-Hip (b) | MK  FA  MD | 0.581 ± 0.064  0.191 ± 0.066  0.986 ± 0.155 | 0.567 ± 0.079  0.184 ± 0.036  1.013 ± 0.137 | 0.610 ± 0.074  0.205 ± 0.030  0.967 ± 0.112 | 3.069  3.016  0.909 | 0.055  0.058  0.407 | 0.144  0.476  0.919 | 0.475  0.664  0.613 | 0.079  0.055  0.606 |
| R-Hip (t) | MK  FA  MD | 0.561 ± 0.066  0.198 ± 0.041  1.124 ± 0.210 | 0.542 ± 0.064  0.179 ± 0.034  1.154 ± 0.184 | 0.582 ± 0.067  0.202 ± 0.044  1.092 ± 0.122 | 2.798  2.920  1.142 | 0.066  0.060  0.326 | 0.298  0.805  0.633 | 0.449  0.097  0.596 | 0.065  0.055  0.097 |
| R-PCC | MK  FA  MD | 0.758 ± 0.107  0.248 ± 0.072  0.901 ± 0.127 | 0.686 ± 0.112  0.220 ± 0.050  0.917 ± 0.086 | 0.773 ± 0.108  0.259 ± 0.056  0.867 ± 0.080 | 5.660  3.593  2.077 | **<0.01**  **<0.05**  0.134 | 0.592  0.514  0.372 | **<0.05**  0.139  0.761 | **<0.01**  **<0.05**  0.053 |
| R-Pr | MK  FA  MD | 0.855 ± 0.119  0.302 ± 0.056  0.887 ± 0.094 | 0.830 ± 0.104  0.292 ± 0.089  0.914 ± 0.122 | 0.923 ± 0.116  0.322 ± 0.063  0.862 ± 0.096 | 4.509  1.492  2.014 | **<0.05**  0.231  0.140 | 0.061  0.624  0.516 | 0.684  0.768  0.602 | **<0.05**  0.135  0.087 |
| R-DT (a) | MK  FA  MD | 0.777 ± 0.098  0.328 ± 0.079  0.997 ± 0.084 | 0.745 ± 0.093  0.315 ± 0.063  1.028 ± 0.096 | 0.816 ± 0.101  0.336 ± 0.049  0.976 ± 0.109 | 4.235  0.817  2.417 | **<0.05**  0.446  0.095 | 0.209  0.735  0.432 | 0.314  0.517  0.224 | **<0.05**  0.136  0.083 |
| R-DT (m) | MK  FA  MD | 0.728 ± 0.117  0.319 ± 0.048  0.981 ± 0.096 | 0.683 ± 0.120  0.299 ± 0.068  1.006 ± 0.084 | 0.675 ± 0.097  0.335 ± 0.064  0.965 ± 0.116 | 2.093  2.925  1.347 | 0.130  0.062  0.266 | 0.431  0.923  0.912 | 0.985  0.581  0.523 | 0.236  0.073  0.116 |
| R-DT (vl) | MK  FA  MD | 0.883 ± 0.094  0.319 ± 0.058  0.912 ± 0.065 | 0.862 ± 0.122  0.302 ± 0.044  0.934 ± 0.102 | 0.914 ± 0.093  0.326 ± 0.037  0.890 ± 0.042 | 2.065  2.996  2.798 | 0.133  0.057  0.066 | 0.533  0.132  0.138 | 1.000  0.919  0.834 | 0.121  0.057  0.054 |
| R-LN | MK  FA  MD | 0.783 ± 0.123  0.259 ± 0.060  0.973 ± 0.086 | 0.770 ± 0.070  0.247 ± 0.042  0.990 ± 0.136 | 0.816 ± 0.125  0.271 ± 0.038  1.041 ± 0.082 | 1.506  2.830  2.263 | 0.229  0.147  0.081 | 0.416  0.513  0.982 | 0.946  0.762  0.078 | 0.137  0.055  0.083 |
| R-CNC | MK  FA  MD | 0.625 ± 0.066  0.243 ± 0.062  0.897 ± 0.149 | 0.613 ± 0.073  0.223 ± 0.037  0.901 ± 0.102 | 0.645 ± 0.102  0.248 ± 0.049  0.852 ± 0.114 | 1.270  2.687  1.516 | 0.286  0.077  0.227 | 0.614  0.421  0.268 | 0.880  0.924  0.999 | 0.293  0.059  0.126 |
| R-FLWM | MK  FA  MD | 0.940 ± 0.120  0.323 ± 0.062  1.032 ± 0.125 | 0.908 ± 0.129  0.313 ± 0.077  1.066 ± 0.216 | 0.974 ± 0.094  0.358 ± 0.068  0.971 ± 0.166 | 2.586  3.744  2.938 | 0.081  **<0.05**  0.060 | 0.615  0.061  0.087 | 0.880  0.584  0.848 | 0.060  **<0.05**  0.063 |
| R-OLWM | MK  FA  MD | 0.969 ± 0.093  0.448 ± 0.066  1.071 ± 0.241 | 0.911 ± 0.117  0.399 ± 0.091  1.095 ± 0.229 | 0.890 ± 0.128  0.450 ± 0.064  0.996 ± 0.108 | 3.646  4.558  3.046 | **<0.05**  **<0.05**  0.057 | 0.059  0.862  0.232 | 0.562  **<0.05**  0.975 | **<0.05**  **<0.05**  0.072 |

*Abbreviations: ECs, elderly controls; SCD plus, subjective cognitive decline plus; aMCI, amnestic mild cognitive impairment; MK, mean kurtosis; FA, fractional anisotropy; MD, mean diffusivity; R-Hip (h), right hippocampus head; R-Hip (b), right hippocampus body; R-Hip (t), right hippocampus tial; R-PCC, right posterior cingulate cortex; R-Pr, right inferior precuneus; R-DT (a), right anterior nucleus of the dorsal thalamus; R-DT (vl), right ventrolateral nucleus of the dorsal thalamus; R-DT (m), right medial nucleus of the dorsal thalamus; R-OLWM, right white matter of the occipital lobe; R-FLWM, right white matter of the frontal lobe; R-LN, right lenticular nucleus; R-CNC, right caput nuclei caudati. Values are presented as the mean ± SD.*

**Supplementary TABLE 4.** Group differences in DKI parameters in left ROIs of ECs, SCD plus and aMCI patients.

| **RIOs** | **ASL**  **metrics** | **Group**  ***SCD plus (n=27) aMCI (n=31) ECs (n=33)*** | | | **F-value ANOVA**  ***p-value*** | | ***Multiple comparison p-value***  ***SCD plus vs ECs SCD plus vs aMCI aMCI vs ECs*** | | |
| --- | --- | --- | --- | --- | --- | --- | --- | --- | --- |
| L-Hip (h) | CBF | 56.550 ± 4.777 | 55.110 ± 6.353 | 59.932 ± 4.361 | 7.155 | **<0.001** | **<0.01** | 0.329 | **<0.001** |
| L-Hip (b) | CBF | 55.060 ± 7.310 | 54.133 ± 7.024 | 57.067 ± 7.356 | 1.378 | 0.257 | 0.444 | 0.626 | 0.323 |
| L-Hip (t) | CBF | 55.118 ± 5.539 | 54.023 ± 5.290 | 56.991 ± 4.546 | 2.715 | 0.072 | 0.247 | 0.446 | 0.059 |
| L-PCC | CBF | 55.069 ± 4.848 | 53.098 ± 6.068 | 58.617 ± 5.558 | 7.895 | **<0.001** | **<0.05** | 0.188 | **<0.01** |
| L-Pr | CBF | 56.012 ± 5.113 | 50.230 ± 4.684 | 52.133 ± 7.429 | 7.025 | **<0.001** | **<0.05** | **<0.01** | 0.227 |
| L-DT (a) | CBF | 63.880 ± 6.429 | 60.671 ± 4.190 | 65.664 ± 8.744 | 4.414 | **<0.05** | 0.367 | **<0.05** | **<0.05** |
| L-DT (m) | CBF | 57.416 ± 5.858 | 55.805 ± 4.509 | 58.958 ± 7.375 | 2.213 | 0.116 | 0.361 | 0.355 | 0.128 |
| L-DT (vl) | CBF | 57.261 ± 5.044 | 56.565 ± 4.245 | 59.029 ± 9.221 | 1.159 | 0.319 | 0.525 | 0.576 | 0.517 |
| L-LN | CBF | 56.263 ± 5.931 | 54.314 ± 6.105 | 57.747 ±5.187 | 2.875 | 0.062 | 0.313 | 0.313 | 0.056 |
| L-CNC | CBF | 59.361 ± 4.908 | 57.099 ± 6.044 | 60.910 ± 5.673 | 3.738 | **<0.05** | 0.262 | 0.182 | **<0.05** |
| L-FLWM | CBF | 24.177 ± 3.040 | 22.812 ± 4.054 | 25.179 ± 4.020 | 3.256 | **<0.05** | 0.276 | 0.149 | **<0.05** |
| L-OLWM | CBF | 24.440 ± 4.434 | 23.279 ± 4.032 | 26.450 ± 4.746 | 4.413 | **<0.05** | 0.144 | 0.305 | **<0.05** |
| R-Hip (h) | CBF | 59.849 ± 6.883 | 57.413 ± 6.673 | 61.555 ± 6.258 | 3.177 | **<0.05** | 0.324 | 0.277 | **<0.05** |
| R-Hip (b) | CBF | 56.699 ± 7.442 | 55.599 ± 6.788 | 59.018 ± 7.883 | 1.378 | 0.257 | 0.371 | 0.561 | 0.202 |
| R-Hip (t) | CBF | 55.514 ± 8.635 | 54.496 ± 5.800 | 57.667 ± 7.927 | 1.474 | 0.235 | 0.222 | 0.748 | 0.169 |
| R-PCC | CBF | 53.917 ± 4.072 | 52.777 ± 4.163 | 56.023 ± 4.212 | 5.054 | **<0.01** | **<0.05** | 0.366 | **<0.01** |
| R-Pr | CBF | 50.471 ± 7.061 | 47.215 ± 5.796 | 52.505 ± 6.849 | 3.314 | **<0.05** | 0.408 | 0.266 | **<0.05** |
| R-DT (a) | CBF | 63.024 ± 6.323 | 61.066 ± 3.945 | 65.431 ± 6.422 | 4.496 | **<0.05** | 0.241 | 0.226 | **<0.01** |
| R-DT (m) | CBF | 58.290 ± 3.261 | 57.485 ± 5.392 | 60.387 ± 5.130 | 3.056 | 0.052 | 0.104 | 0.509 | 0.096 |
| R-DT (vl) | CBF | 57.087 ± 5.945 | 55.558 ± 4.017 | 58.848 ± 6.885 | 2.615 | 0.079 | 0.296 | 0.439 | 0.068 |
| R-LN | CBF | 56.263 ± 5.931 | 54.314 ± 6.105 | 57.747 ±5.187 | 1.299 | 0.278 | 0.538 | 0.559 | 0.357 |
| R-CNC | CBF | 62.777 ± 8.192 | 58.844 ± 5.982 | 63.125 ± 8.616 | 2.944 | 0.060 | 0.874 | 0.079 | 0.051 |
| R-FLWM | CBF | 24.379 ± 2.989 | 23.304 ± 4.324 | 24.912 ± 3.318 | 1.658 | 0.197 | 0.516 | 0.406 | 0.306 |
| R-OLWM | CBF | 25.566 ± 3.223 | 23.742 ± 4.147 | 26.898 ± 6.321 | 3.408 | **<0.05** | 0.876 | 0.106 | 0.063 |

*Abbreviations: ECs, elderly controls; SCD plus, subjective cognitive decline plus; aMCI, amnestic mild cognitive impairment; CBF, cerebral blood flow; Hip (h), hippocampus head; Hip (b), hippocampus body; Hip (t), hippocampus tial; PCC, posterior cingulate cortex; Pr, inferior precuneus; DT (a), anterior nucleus of the dorsal thalamus; DT (vl), ventrolateral nucleus of the dorsal thalamus; DT (m), medial nucleus of the dorsal thalamus; OLWM, white matter of the occipital lobe; FLWM, white matter of the frontal lobe; LN, lenticular nucleus; CNC, caput nuclei caudati.*
